# Flexible bronchoscopic management of benign tracheal stenosis: long term follow-up of 115 patients

**DOI:** 10.1186/1749-8090-5-2

**Published:** 2010-01-17

**Authors:** Nader Abdel Rahman, Oren Fruchter, David Shitrit, Benjamin D Fox, Mordechai R Kramer

**Affiliations:** 1 The Pulmonary Institute, Rabin Medical Center, Beilinson Campus, Petah Tiqwa, Israel

## Abstract

**Background:**

Management of benign tracheal stenosis (BTS) varies with the type and extent of the disease and influenced by the patient's age and general health status, hence we sought to investigate the long-term outcome of patients with BTS that underwent minimally invasive bronchoscopic treatment.

**Methods:**

Patients with symptomatic BTS were treated with flexible bronchoscopy therapeutic modalities that included the following: balloon dilatation, laser photo-resection, self-expanding metal stent placement, and High-dose rate endobronchial brachytherapy used in cases of refractory stent-related granulation tissue formation.

**Results:**

A total of 115 patients with BTS and various cardiac and respiratory co-morbidities with a mean age of 61 (range 40-88) were treated between January 2001 and January 2009. The underlining etiologies for BTS were post - endotracheal intubation (N = 76) post-tracheostomy (N = 30), Wegener's granulomatosis (N = 2), sarcoidosis (N = 2), amyloidosis (N = 2) and idiopathic BTS (N = 3). The modalities used were: balloon dilatation and laser treatment (N = 98). Stent was placed in 33 patients of whom 28 also underwent brachytherapy. Complications were minor and mostly included granulation tissue formation. The overall success rate was 87%. Over a median follow-up of 51 months (range 10-100 months), 30 patients (26%) died, mostly due to exacerbation of their underlying conditions.

**Conclusions:**

BTS in elderly patients with co-morbidities can be safely and effectively treated by flexible bronchoscopic treatment modalities. The use of HDR brachytherapy to treat granulation tissue formation following successful airway restoration is promising.

## Introduction

The most common etiology for acquired benign tracheal stenosis (BTS) is tracheal intubation (even for a short period of time) or tracheostomy [1,2]. Acquired BTS may also occur due to traumatic intubation, laryngeal trauma due to previous laryngeal surgery and prior surgery for upper airway papillomatosis. BTS can also be accidental as a result of inhalational (thermal or caustic) injury or complicating cervical trauma (either blunt or penetrating). Rare causes for BTS include autoimmune diseases as well as inflammatory diseases such as Wegener's granulomatosis, sarcoidosis and systemic lupus erythromatosis [3].

The management of BTS varies with the type and extent of the disease and depends upon the age and co-morbidities of the patient [4]. The optimal therapeutic approach for BTS is surgical reconstruction [5,6]. However many elderly patient with underlying conditions are inoperable and require endobronchial palliation. Treatment options include laser ablation of granulation tissue, electro coagulation, mechanical dilatation, and/or stent placement into the affected area [7-15].

The aim of the current report was to review our experience in treating BTS secondary to various etiologies, review the treatment strategies used, and evaluate long-term success rates and complications.

## Patients and methods

Our institute serves as a regional referral center for interventional bronchoscopy procedures. We retrospectively analyzed the medical records of all patients who were refereed to our institute for evaluation and management of symptomatic BTS between January 2001 and January 2009. Institutional review board approval was obtained, but specific informed consent was not required for this retrospective study. Informed consent for each bronchoscopy was obtained prior to the procedure.

Each patient underwent a standard pre-operative assessment, including physical examination, routine laboratory tests, spirometry, chest radiography and computed tomography of the chest. An initial diagnostic flexible bronchoscopy was performed for each patient to identify the type, location and severity of the stenosis. The degree of stenosis was estimated visually. Flexible bronchoscopy was performed under conscious sedation with midazolam (1-10 mg) and alfentanyl (0. 5 mg-1.5 mg), in the bronchoscopy suite and nder Spontaneous ventilation with supplemental O2 through nasal canula. The equipment used included flexible bronchoscopes (Pentax Co. Tokyo, Japan or Olympus Co. Tokyo, Japan). For web-like stenoses a recommended mucosal sparring technique with radial incisions followed by airway dilatation using balloon bronchoplasty as described by Mehta [9]. For stenosis produced by granulation tissue formation around the tracheal stoma, photocoagulation with Nd:YAG laser was used. Stent implantation (self-expanding non-covered metal stent (SMART nitinol stent, Cordis, Miami,. FL, USA or Wallstent Boston Scientific Corp; Natick, MA, USA) was used in patients with recurrent stenosis. Brachytherapy was used when a patient required 3 or more interventions within 6 months due to recurrent granulation tissue formation In cases of recurrent refractory granulation tissue around a metal stent placed for restoration of airway patency, High-dose rate (HDR) endobronchial brachytherapy and single application of a total 10 Gy was administrated along the stent using a brachytherapy remote afterloader with a ^192^Ir source as previously described [16].

If re-stenosis occurred on a follow up bronchoscopy (usually every 4 to 6 weeks for the first 6 months) then another intervention was applied.

Patients were considered cured when free of symptoms for at least one year after the last interventional procedure. Statistical analysis Descriptive data are presented as mean (± SD) or median (range). Survival curves were evaluated by the Kaplan-Meier method. Statistical analyses were performed using a statistical software package (MedCalc Version 9.3.0.0, USA).

## Results

**Figure 1:**
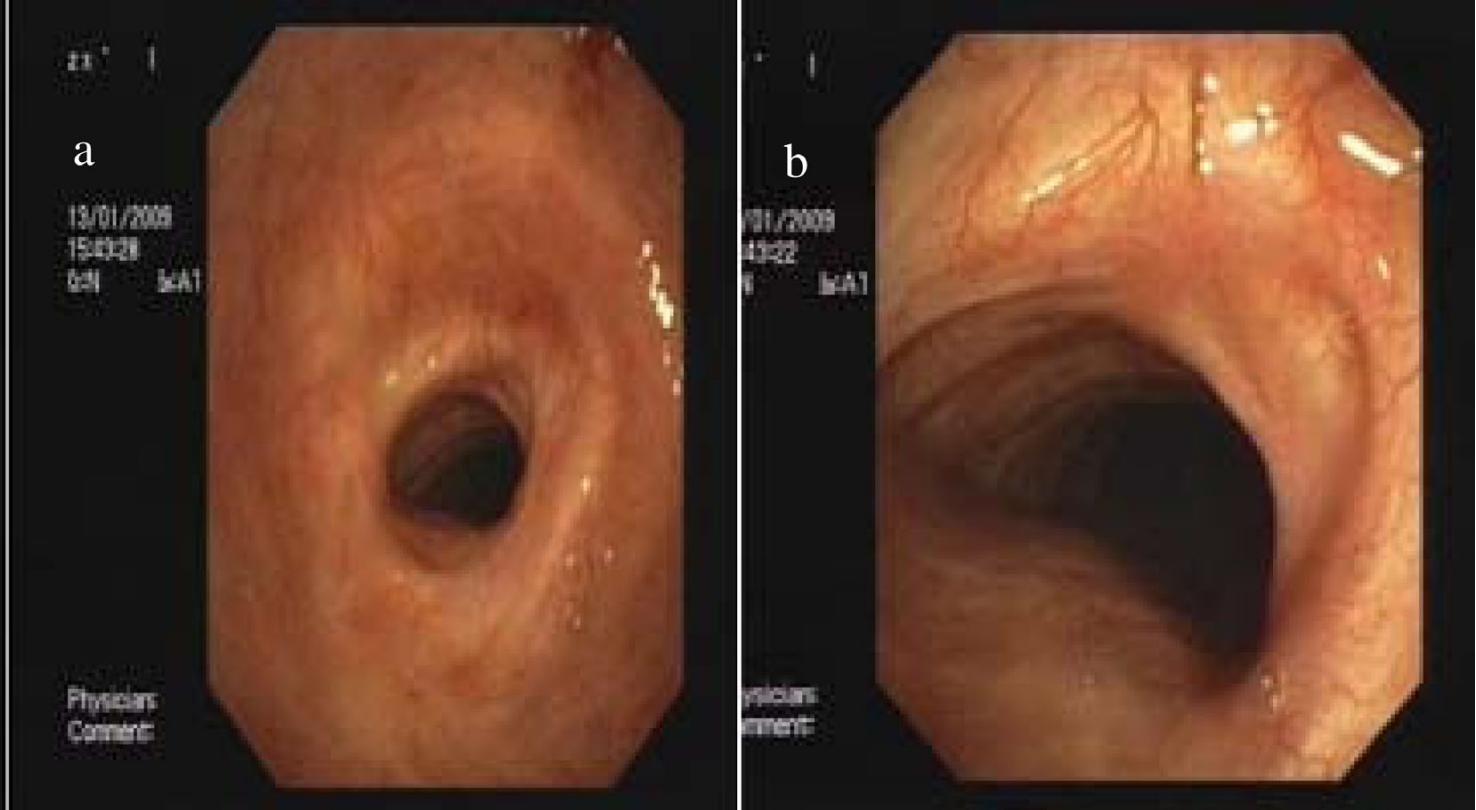
**Post-intubation benign tracheal stenosis in a 78-year old patient who was mechanically ventilated for 10 days due to chronic-obstructive lung disease**. Concentric web-like tracheal obstruction before (a) and after (b) Nd-YAG laser resection and balloon dilatation.

**Figure 2:**
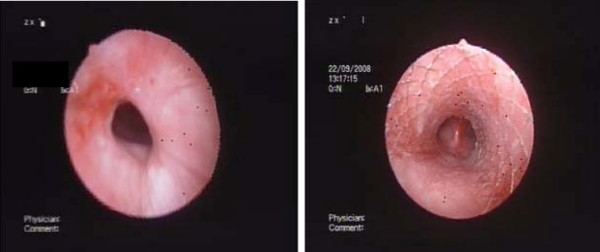
**Post-tracheostomy benign tracheal stenosis in a 56-year old female patient who was mechanically ventilated for 67 days following multiple trauma**. Concentric web-like tracheal obstruction before (a) and after (b) Nd-YAG laser resection balloon dilatation, and self-expanding metallic stent placement.

A total of 115 patients (65 males and 50 women) with a median age of 61 (range 40-88) Were identified between January 2001 and January 2009. All patients presented with signs and symptoms of upper airway obstruction including shortness of breath, stridor, cough, dyspnea and wheezing, and presented with typical flow volume curve that demonstrate fixed airway obstruction The etiologies for BTS are presented on Table 1. The vast majority of patients had BTS following mechanical ventilation through endotracheal tube (N = 76) or tracheostomy (N = 30). The remaining patients had Wegener's granulomatosis, Sarcoidosis, amyloidosis and idiopathic BTS. The morphologic characteristics of the lesions are described on Table 2. Most patients had significant co-morbidities and the most common were congestive heart failure (N = 36) and chronic obstructive lung disease (N = 34), nine patients had non-respiratory tract malignancy. A total of 760 endoscopic procedures (a mean of 6 procedures per patient) were performed. The median duration of the procedure was 37 minutes( range 14-45 minutes). All patients underwent balloon dilatation as an initial temporary relieving procedure. There were 460 sessions of laser treatment in 98 patients, of whom 58 patients required only laser photo-resection without a need for another treatment modalities (Figure 1). A total of 33 patients, all of them had BTS due to previous mechanical ventilation through endotracheal tube, underwent stent insertion (Figure 2), of whom 28 underwent brachytherapy for prevention of granulation tissue re-formation. All patients experienced a reduction in therapeutic bronchoscopic procedures after HDR brachytherapy compared with the pretreatment period, there were no treatment related complications. All 85 patients who presented without post-tracheostomy stenosis reported subjective relief. Out of 30 patients who presented with post-tracheostomy tracheal stenosis, 15 (50%) underwent successful de-cannulation. The overall success rate was 87%.

**Table 1:** Underlining etiologies for benign tracheal stenosis among study subjects

POST MECHANICAL VENTILATION (N = 106)	OTHER ETIOLOGIES (N = 9)
CHF (24)	Sarcoidosis (1)
COPD (22)	WG (2)
COPD & CHF (12)	Amyloidosis (1)
Multiple trauma (11)	Post laryngeal surgery (1)
Pneumonia (10)	Idiopathic (4)
Malignancy (9)	
Other (18)	

Abbreviations: CHF: congestive heart failure; COPD:chronic obstructive lung disease; WG: Wegener's granulomatosis

**Table 2:** The morphologic characteristics of the tracheal lesion treated

LOCATION	TYPE	LENGTH	DEGREE OF STENOSIS
Subglottic (81)	Web-like (97)	< 1 cm (12)	< 30% (4)
			

Upper trachea (30)	Complex (18)	1-3 cm (99)	30-50% (57)
Mid and lower trachea (4)		> 3 cm (4)	50-99%(56)

**Figure 3:**
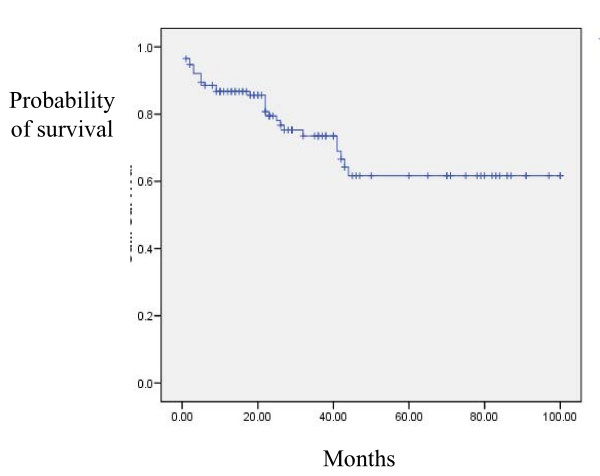
**Kaplan-Meyer survival analysis of study patients following the first bronchoscopic intervention**.

There were no intraoperative and peri-operative deaths. Procedure complications were relatively minor and manageable and included granulation tissue formation that required recurrent laser treatments (N = 30), and tracheoesophegial fistula (N = 1). We obsereved no other complication as stent fracture or a patient in whom replacement of the stent was required. Over a median follow-up of 51 months (range 10-100 months), 30 patients (26%) died. Timing from the first intervention to death ranged from 1 day to 6 years. The causes of death were: Sepsis (N = 24), CHF (N = 8), bronchopleural fistula (N = 1), COPD (N = 3), pneumonia (N = 3), and malignancy (N = 4). As can be seen, most death cases were due to exacerbation of the underlying condition and not directly related to the interventional procedure. Cumulative survival rates at 1, 2, and 5 years were 80%, 73%, and 62% respectively (Figure 3).

## Discussion

Surgical resection and end-to-end anastomosis is still considered to be the optimal treatment for a young patient presenting with symptomatic BTS [5,6]. For elderly subjects or for patients with significant underlining co-morbidities, endoscopic treatment should be considered [4]. In the current large case series we have shown that bronchoscopic treatment modalities can provide durable successful results in selected patients with BTS with a relatively low rate of complications. Previous works [7-15] have discussed the bronchoscopic management of BTS and reported similar success rates. Similar to a previous smaller report [8], we noted that BTS after endotracheal intubation and after tracheostomy differ in the success rates of airway restoration. The relatively low success rate of de-cannulation reflects the different pathophysiological mechanism for their formation [8]. BTS that occurs at the endotracheal tube cuff site appears as web-like fibrous growth, due to Ischemic damage and fibrotic healing process. On the other hand, BTS following tracheostomy often involves apart from granulation tissue formation also cartilage damage and malacia. This difference may account for the difference in the excellent results we obtained in post -intubation BTS and the relatively disappointing outcome in post-tracheostomy BTS. Another explanation lies in the notion that patients who could not be weaned from mechanical ventilation and had to be switched from endotracheal tube to tracheostomy represent sub-group of patients who are considerably sicker. The difficulties in de-cannulation following endobronchial treatment also stem fron their general health condition and co-morbidities.

Our case series is different from previous works in several aspects that should be addressed. First, we employed only flexible bronchoscope-based modalities and did not use as in most previous works [3,4,8-15], rigid bronchoscopy. The advantages of rigid bronchoscopy in treating BTS are well known and include airway safety, the ability to perform mechanical debulking, and the ease of blood and airway secretion cleaning. The major drawbacks are the need for operating room, anesthesiologist. and general anesthesia that are obviated by the use of flexible bronchoscopy under conscious sedation. We observed no major intra-operative complication and feel that BTS treatment can be safely and cost-effectively carried out in the bronchoscopy suite by an experienced team. Given the fact that all patients in whom a stent was required had sub-glottic stenosis we preferred not to use silicone stents. When a straight-type Dumon silicone stent is placed in the subglottic trachea, movement of the head and neck or coughing tend to induce stent migration [3]. The other unique aspect of the current report is the use of HDR brachytherapy to treat and prevent recurrent refractory granulation tissue formation around a metal stent placed for restoration of airway patency following lumen restoration in BTS. Previous works focused on the use of corticosteroids injections and Mitomycin-C applications for treatment of reccurent refractory granulation tissue formation and stenosis following treatment of BTS are [3,4]. HDR endobronchial brachytherapy is widely used as a palliative measure to overcome mechanical obstruction of major airways caused by either primary or secondary malignant tumors. It allows the delivery of a high dose of radiation over a short period of time to the obstructed area, and at the same time reducing the risk of undesired side effects to adjacent structures. Our group was the first [16] followed by others [17] to describe the use of High-dose rate (HDR) endobronchial brachytherapy to prevent and treat stent granulation in benign conditions. In the current series HDR brachytherapy was successfully employed in 28 patients who had refractory metal stent stenosis due to granulation tissue formation. underwent brachytherapy for prevention of granulation tissue re-formation. As more experience is gained with this approach, we believe that the use of endobronchial HDR brachytherapy will widely expand. The procedure is safe, particularly in patients for whom surgical mortality is by far more harmful. We believe that this method should be used whenever a metal stent placed for BTS is complicated by recurrent granulation tissue growth.

## Conclusion

Most cases of BTS in elderly subjects with co-morbidities can be safely and effectively treated by flexible bronchoscopic treatment modalities. The use of HDR brachytherapy to prevent and treat granulation tissue formation following successful airway restoration in these cases is promising but should be further explored.

## Competing interests

The authors declare that they have no competing interests.

## Authors' contributions

NAR, OF, DS, BJF, MRK carried out the clinical work, drafted the manuscript and participated in its design. All authors read and approved the final manuscript.
